# A polyextremophilic alcohol dehydrogenase from the Atlantis II Deep Red Sea brine pool

**DOI:** 10.1002/2211-5463.12557

**Published:** 2018-12-18

**Authors:** Anastassja L. Akal, Ram Karan, Adrian Hohl, Intikhab Alam, Malvina Vogler, Stefan W. Grötzinger, Jörg Eppinger, Magnus Rueping

**Affiliations:** ^1^ KAUST Catalysis Center (KCC) King Abdullah University of Science and Technology (KAUST) Thuwal Saudi Arabia; ^2^ Center for Integrated Protein Science Munich at the Department of Chemistry Technical University of Munich (TUM) Garching Germany; ^3^ Computational Bioscience Research Center (CBRC) King Abdullah University of Science and Technology (KAUST) Thuwal Saudi Arabia; ^4^ Institute of Biochemical Engineering Technical University of Munich (TUM) Garching Germany; ^5^ Institute of Organic Chemistry RWTH Aachen Aachen Germany

**Keywords:** alcohol dehydrogenase, extremophiles, extremozyme, halophiles, thermophiles

## Abstract

Enzymes originating from hostile environments offer exceptional stability under industrial conditions and are therefore highly in demand. Using single‐cell genome data, we identified the alcohol dehydrogenase (ADH) gene, *adh/a1a*, from the Atlantis II Deep Red Sea brine pool. ADH/A1a is highly active at elevated temperatures and high salt concentrations (optima at 70 °C and 4 m KCl) and withstands organic solvents. The polyextremophilic ADH/A1a exhibits a broad substrate scope including aliphatic and aromatic alcohols and is able to reduce cinnamyl‐methyl‐ketone and raspberry ketone in the reverse reaction, making it a possible candidate for the production of chiral compounds. Here, we report the affiliation of ADH/A1a to a rare enzyme family of microbial cinnamyl alcohol dehydrogenases and explain unique structural features for halo‐ and thermoadaptation.

**Abbreviations**
ADH: alcohol dehydrogenase
CAD: cinnamyl alcohol dehydrogenase
CDD: conserved domain database
MDR: medium‐chain dehydrogenase/reductase
SAG: single amplified genome

Alcohol dehydrogenases (ADHs, EC1.1.1.1) are enzymes that catalyze the reversible dehydrogenation of alcohols to aldehydes or ketones by consuming the cofactor nicotinamide adenine dinucleotide or its phosphate (NAD^+^/NADP^+^). In the biochemical industry, the natural regio‐ and enantioselectivity of ADHs are exploited to produce chiral compounds for pharmaceuticals and fine chemicals and are therefore worthwhile studying 1, 2. One of the major drawbacks of conventional ADHs is their poor performance under industrial conditions such as extreme temperatures, pH, and the presence of salts and organic solvents, as they tend to lose their activity and stability 3. However, these operational conditions are a prerequisite to increase the solubility of hydrophobic compounds, reduce viscosity and accelerate the reaction, or influence the enantioselectivity 4, 5. Besides the catalytic reaction, the enzymes have to endure unnatural conditions during transportation, storage, and formulation 2. Therefore, industry demands the development of more robust and stereoselective ADHs.

In addition to protein‐engineering methods, the exploration of enzymes derived from extremophilic organisms is a major pool for highly stable ADHs 6. Extremophiles are naturally adapted to survive and grow in hostile environments, and their evolved enzymes, also called extremozymes, are not only stable but highly active under extreme conditions 7. The structurally promoted resistance of extremozymes is still poorly understood and currently under investigation 8.

Extremozymes derived from marine microorganisms are reported to be stable under harsh conditions including high ionic strengths and solvents and further show novel functionalities 9. One of the most extreme marine environments is the Red Sea brine pools, characterized by high salinity, elevated temperatures, high concentrations of heavy metals, hydrostatic pressure, and lack of oxygen and light 10. Several enzymes derived from brine pools exhibit polyextremophilic characteristics and are therefore worth to be studied to elucidate their structure–stability relationship and to find potential enzymes for industrial applications 11, 12.

For the discovery of novel extremozymes, we explored the largest Red Sea brine pool, the Atlantis II Deep, which exhibits temperatures up to 68 °C and salinities up to 26%. Several genes were annotated from single amplified genomes (SAGs) of uncultured single cells derived from water samples and are open for exploration 13. Here, we characterized the ADH gene, *adh/a1a*, from an uncharacterized *MSBL1 archaeon* collected from the Atlantis II Deep interphase (2036 m, 16.8% salt, 63 °C, pH 5.3). ADH/A1a was overexpressed in the halophilic *Haloferax volcanii* strain under high‐salt condition, resembling its natural environment. The purified ADH/A1 was highly active in high salt concentrations and elevated temperatures and resistant toward organic solvent additives. Furthermore, ADH/A1a revealed to have a broad substrate spectrum, with a preference for primary long‐chain and aromatic alcohols such as cinnamyl alcohol and its derivatives. The *in silico* structural analysis of ADH/A1a and homologs uncovered the evolved structural features of its halo‐thermophilic adaptation and may lead to engineering strategies for robust and rational designed biocatalysts. The phylogenetic analysis revealed that ADH/A1a shares features of classical cinnamyl alcohol dehydrogenases (CADs) found in plants and propanol‐preferring ADHs 14, 15.

## Materials and methods

### Chemicals, strains, and vectors

The restriction endonucleases and chemically competent *Escherichia coli* cells were purchased from New England Biolabs (Ipswich, MA, USA). Most other chemicals were purchased from Sigma (St. Louis, MO, USA). *Haloferax volcanii* H1895 and its corresponding vector pTA963 were kindly provided by Thorsten Allers (Institute of Genetics, University of Nottingham, Queen's Medical Centre, UK) and Eva Strillinger (Institute of Biochemical Engineering, TUM, Germany) 16, 17.

### Source and annotation of SAGs from the Red Sea brine pools

Samples were collected from different depths of several Red Sea brine pools. From these samples, single cells were sorted and lysed, and the whole genome was amplified and sequenced 18. The SAGs were annotated and the putative genes were fed into the INDIGO database 13. The gene *adh/a1* was derived from the SAG of an unclassified *MSBL1* (Mediterranean Sea Brine Lake 1) archaeon of the Atlantis II Deep interphase and identified by the profile pattern match algorithm 19.

### Construction of expression plasmid pTA963‐*adh/a1a*

The *adh/a1* gene was codon‐optimized using the Java codon adaptation online tool (JCat) for the codon usage of *Halobacterium* sp. NRC‐1, which is similar to the codon usage of *H. volcanii* 20, 21. The gene was synthesized by Geneart (Regensburg, Germany) and cloned into the plasmid pTA963. The shortened gene *adh/a1a*, which excluded the N‐terminal nonsense sequence of *adh/a1*, was created using the Gibson assembly cloning kit from NEB (Ipswich, MA, USA). The cloned construct pTA963‐*adh/a1a* was transformed into the biofilm‐free expression strain *H. volcanii* H1895 using the protocol from the Halohandbook 22. The shortened gene *adh/a1a* was deposited in National Center for Biotechnology Informations (NCBI's) GenBank (GenBank: KXB02677) 18.

### Production and purification of ADH/A1a

Protein production in *H. volcanii* and purification using a HisTrap HP column (GE Healthcare, Little Chalfont, UK) were performed as described with some buffer modifications 17. The running and storage buffer contained additionally 10% (v/v) of glycerol (running/storage buffer: 10 mm HEPES/NaOH, pH 7.5, 2 m NaCl, 10% v/v glycerol). After purification, the active fractions were concentrated (~ 2–3 mg·mL^−1^) using an Amicon Ultracel^®^ 30 000 MWCO (Millipore, Billerica, MA, USA).

For further purification, the concentrated protein solution was loaded onto a HiLoad 16/60 Superdex 75 prep grade preparative column (GE Healthcare) with a flow rate of 1 mL·min^−1^. The active protein fractions were analyzed by sodium dodecyl sulfate/polyacrylamide gel electrophoresis (SDS/PAGE), and the purity of the protein was calculated using imagej version 1.51 (Wayne Rasband; NIH, Bethesda, MD, USA). The protein concentration was measured using a NanoDrop 2000c spectrophotometer (Thermo Scientific, Waltham, MA, USA). The extinction coefficient (26 930 m ^−1^·cm^−1^) and molecular weight (37.6 kDa) of ADH/A1a were calculated using ProtParam 23.

### Tryptic digest and LC‐MS/MS analysis of ADH/A1a

The identification of ADH/A1a was performed by LC‐MS/MS analysis, using a Coomassie‐stained protein band, excised from a SDS/PAGE, destained, and digested using the In‐Gel Tryptic Digest Kit (Thermo Fisher Scientific, Waltham, MA, USA). Peptides were measured using a LTQ Orbitrap mass spectrometer (Thermo Fisher Scientific) and analyzed using mascot v2.3 (Matrix Sciences Ltd, Manchester, UK).

### Determination of ADH/A1a activity

The activity of ADH/A1a was determined by measuring the absorbance of NAD(P)H at 340 nm with a spectrophotometer (Cary 60; Agilent, Santa Clara, CA, USA). The reaction was performed in 1‐mL semi‐micro PMMA cuvettes (Sigma‐Aldrich) at 60 °C for 30 min. Unless otherwise specified, the oxidation reaction was performed in 3 m KCl, 50 mm glycine/NaOH buffer (pH 10.0) with 10 mm NAD(P)^+^, and 0.2% (v/v) substrate; the reduction reaction was performed in 3 m KCl, 50 mm K_2_HPO_4_/citric acid buffer (pH 6.0) with 0.2 mm NAD(P)H, and 0.4% (v/v) substrate. Before the reaction, the ADH/A1a (0.4–1 μm) was incubated with zinc sulfate (0.4 mm) in the reaction mixture at room temperature for 15 min. The reaction was induced by the addition of the substrate. The kinetic parameters were calculated with graphpad prism v7 (GraphPad Software Inc., La Jolla, CA, USA).

### Thermal stability of ADH/A1a

The thermal melting curve was determined using differential static light scattering measured with a Stargazer‐2 (Harbinger Biotechnology, Toronto, ON, Canada) with a 1.5 mg·mL^−1^ ADH/A1a sample in dialysis buffer (10 mm HEPES/NaOH, pH 7.5, 2 m NaCl). The enzyme was pre‐incubated with zinc sulfate for 30 min and dialyzed against dialysis buffer for 2 h at RT before measurement. The melting temperature *T*_m_ was calculated using graphpad prism (GraphPad Software Inc.).

### Effect of various parameters on the ADH/A1a activity

The effect of various metal ions was tested with metal‐free ADH/A1a. To remove nonspecific bound metal ions, the protein was dialyzed overnight at 4 °C against dialysis buffer containing 10 mm EDTA. Afterward, EDTA was removed by further dialysis against EDTA‐free dialysis buffer. ADH/A1a was incubated with the metal ions for 30 min at room temperature before reaction.

Different NAD(P)^+^ concentrations were tested in the oxidation reaction. The reaction was performed under standard conditions (60 °C, 3 m KCl, pH 10) using cinnamyl alcohol as substrate.

### Zinc content of active ADH/A1a solution

An ADH/A1a solution (*c* = 1.7 mg·mL^−1^, *V* = 1 mL) was incubated with 20 μL of 100 mm ZnSO_4_ solution for 1 h at room temperature and subsequently dialyzed against dialysis buffer (3 × buffer exchange) at 4 °C for 24 h. The samples were recorded with a 5100 ICP‐OES instrument (Agilent) coupled to an SPS 4 Autosampler (Agilent), using argon as gas supply. The digestion was done with 8 mL of nitric acid at 240 °C and 100 bar using an UltraWAVE apparatus (Milestone, Shelton, CT, USA). Prior to digestion of the samples, vessel cleaning was performed using only nitric acid (5 mL). All the samples and measurements were taken in duplicates.

### Bioinformatic analysis

The analysis of the amino acid sequence and the classification of the protein family and subfamily were done using the CD‐search tool of the Conserved Domain Database (CDD) of the NCBI 14, 24. The subdomains were identified using interproscan 25. The secondary structure elements of ADH/A1a were predicted using jpred 4 26. Sequence‐based homologous ADHs with resolved crystal structures were identified using SWISS‐MODEL and I‐TASSER 27, 28. The homology model was created using SWISS‐MODEL and was evaluated by the GMQE and QMEAN scores 29. The QMEANDisCo method was used to validate the local quality of the model 30. The electrostatic surface potential was calculated using APBS of the PDB2PQR 2.0 with the default values and applied to the structures using the APBS plugin of pymol v1.7 (Schrödinger LLC, New York, NY, USA) 31, 32. The surface‐accessible residues were calculated with the Swiss‐PDB Viewer 4.1.1 using ≥ 30% surface accessibility 33.

For the phylogenetic analysis, experimentally confirmed ADH sequences of various subfamilies were selected using UniProt 34. Uncharacterized sequences of the CAD2 were selected from the CDD 24, 35. Sequence alignments were done using mafft version 7 34. The phylogenetic tree was constructed with FastTree2 using the maximum‐likelihood method and the Jones–Taylor–Thornton substitution model (1000 bootstraps, default settings) 36. Functional annotation and possible metabolic pathways were investigated using BlastKOALA of the kegg 37.

## Results

### Analysis of *adh/a1a*

The gene *adh/a1* was annotated from a single amplified genome (SAG) of an uncultured *MSBL1* archaeon, which was derived from the Atlantis II Deep Red Sea brine pool 13. Our analysis of the gene sequence revealed the rare alternative start codon UUG, which was previously missed by the annotation algorithm and is known to be used by haloarchaea 38. Therefore, we deleted the N‐terminal 20‐amino acid‐long random coil region of *adh/a1* and named the shortened gene *adh/a1a*. We classified ADH/A1a as a member of the zinc‐binding medium‐chain alcohol dehydrogenase (MDR) superfamily, exhibiting highly conserved domains of the CAD family 15. Based on the conserved features of the CAD family, the catalytic and structural zinc‐binding sites, as well as putative NAD(P)H‐ and substrate‐binding sites, were predicted. Furthermore, the N‐terminal GroES‐like domain (position 1–152) and the C‐terminal NAD(P)‐binding domain (position 153–294) were identified (Fig. S1).

### Production and purification of ADH/A1a

Initial attempts to produce ADH/A1a in *E. coli* under low‐salt conditions resulted in an insoluble and inactive protein aggregate (data not shown). Therefore, ADH/A1a was expressed in the halophilic *H. volcanii* H1895 expression system with an N‐terminal his_6_‐tag 17. The expression and purification in the presence of high salt concentrations resulted in soluble and active ADH/A1a (~ 15 mg L^−1^ yield; Figs S2 and S3A). In contrast, purification at a low salt concentration (≤ 0.5 m NaCl) resulted in inactivation of the enzyme; however, it increased the purity (Fig. S3B). The native PAGE analysis revealed the oligomeric state of the enzyme (Fig. S4). The identity of the ADH/A1a and residual proteins was confirmed by tryptic digest and LC‐MS/MS analysis (Fig. S5, Table S1).

### Characterization of ADH/A1a

The deletion of the N‐terminal nonsense region of the ADH/A1 did not affect the activity or stability of the ADH (data not shown). Therefore, the correct annotated ADH/A1a was used for characterization.

**Figure 1:**
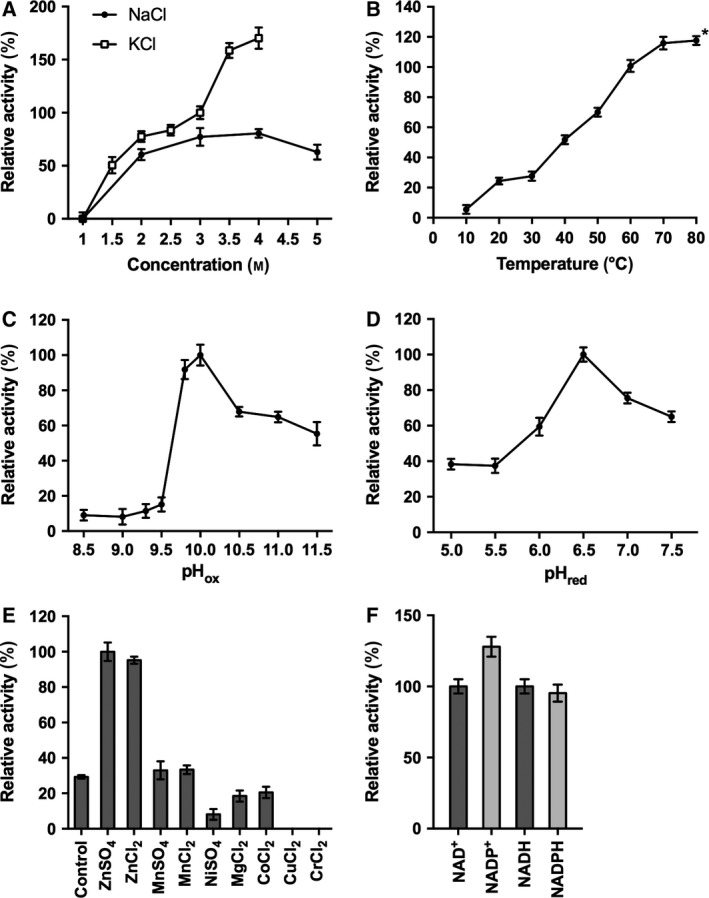
Effect of various factors on the enzyme activity. (A) Salt concentration (saturation limit: 4 m for KCl, 5 m for NaCl), (B) temperature, (C) pH in oxidation, (D) pH in reduction reaction, and (E) metal ions (0.2 mm), protein without additive as control; and (F) cofactors for oxidation/reduction (oxidation: 100% = 0.49 μmol·min^−1^·mg^−1^; reduction: 100% = 0.31 μmol·min^−1^·mg^−1^). Error bars indicate SDs. *Protein precipitated during the reaction.

The activity of the purified ADH/A1a was assayed over a broad range of salt (NaCl and KCl) concentrations, temperatures, and pH values to determine the optimal reaction conditions. ADH/A1a was active in a salt concentration range of 1.5–5 m, with the maximum activity found at 4 m salt; higher activities were observed in KCl than in NaCl (Fig. 1A). The effect of temperature on the enzyme activity was assayed from 10 to 80 °C, with activity peaking at the relatively high temperature of 70 °C (Fig. 1B). Substantial activity was observed at 80 °C; however, over time protein aggregation occurred that is in agreement with the determined *T*_m_ of ~ 73 °C (Fig. S6). The enzyme showed very divergent pH activity optima in the oxidation and reduction reactions, at pH 10.0 and pH 6.5, respectively (Fig. 1C,D). A thermodynamic analysis of the reactions revealed concordantly that the oxidation reaction is energetically favored at a basic pH, whereas the reduction reaction is favored at an acidic pH (Fig. S7; calculated with eQuilibrator 39).

The catalytically active metal was determined by incubating ADH/A1a with different metal ions before catalysis. The enzyme activity increased 3.5‐fold with the addition of zinc(II) ions. In contrast, manganese(II) had no effect, and nickel(II), magnesium(II), and cobalt(II) had adverse effects on the enzyme activity. The addition of either copper(II) or chromium(II) resulted in a total loss of enzyme activity (Fig. 1E). Metal‐free ADH/A1a showed activity exclusively with zinc(II), confirming its zinc dependency (Fig. S8A). Relatively high zinc concentrations (≥ 0.1 mm) were required in the reaction buffer to reach the optimal activity of the enzyme (Fig. S8B). Furthermore, a high content of bound zinc ions (18.9 g‐atoms per ADH/A1a subunit) was determined in the ADH/A1a solution by ICP‐OES (Table S2).

The cofactors NAD^+^/NADH and NADP^+^/NADPH were almost equally accepted (Fig. 1F). The optimal cofactor concentration for the oxidation reaction was 10 mm, showing substrate inhibition with higher concentrations (Fig. S8C).

Alcohol dehydrogenase/A1a oxidized a broad range of substrates including small‐, medium‐, and long‐chain alcohols with a preference for long primary alcohols (Table 1A). Notably, several phenol and terpene alcohols were accepted as substrate. The highest reaction rates were achieved with cinnamyl alcohol (~ 0.49 μmol·min^−1^·mg^−1^), followed by 1,5‐pentanediol and 1‐heptanol (~ 0.29 μmol·min^−1^·mg^−1^ for both). Remarkably, the secondary alcohol 3‐buten‐2‐ol (~ 0.05 μmol·min^−1^·mg^−1^) was also oxidized, though at slow rates. In the reduction reaction, a conversion was exclusively found for cinnamaldehyde (0.31 μmol·min^−1^·mg^−1^), raspberry ketone (~0.25 μmol·min^−1^·mg^−1^), and cinnamyl‐methyl‐ketone (~0.40 μmol·min^−1^·mg^−1^; Table 1B).

**Table 1:** Substrate scope of ADH/A1a

Substrate	Relative activity (%)
(A) Substrates (0.2% v/v) in the oxidation reaction (100% = 0.49 μmol·min^−1^·mg^−1^)	
Ethanol	15.2 ± 2.6
1‐Propanol	11.9 ± 4.1
3‐Buten‐2‐ol	9.2 ± 2.0
Benzyl alcohol	26.9 ± 2.0
Isoamyl alcohol	11.6 ± 2.1
Prenol	29.2 ± 2.0
1‐Butanol	32.3 ± 5.5
1‐Pentanol	46.5 ± 6.1
1,5‐Pentanediol	59.6 ± 3.0
1‐Heptanol	59.1 ± 1.2
Hydrocinnamyl alcohol	53.7 ± 1.6
Cinnamyl alcohol	100.0 ± 5.0
Geraniol	36.1 ± 2.4
Citronellol	33.5 ± 3.4
Nerol	14.9 ± 2.4
(B) Substrates (0.4% v/v) in the reduction reaction (100% = 0.40 μmol·min^−1^·mg^−1^)	
Cinnamaldehyde	76.6 ± 4.3
Raspberry ketone	63.4 ± 5.2
Cinnamyl‐methyl‐ketone	100.0 ± 2.2

Alcohol dehydrogenase/A1a followed Michaelis–Menten kinetics in the oxidation reaction and showed a first‐order reaction (Fig. S9). The kinetic parameters confirmed the preference for cinnamyl alcohol as a substrate (Table 2).

**Table 2:** Kinetic parameters of ADH/A1a

Substrate	*V*_max_ (mU·mg^−1^)	*K*_m_ (mm)	*k*_cat_ (s^−1^)	*k*_cat_/*K*_m_ (s^−1·^mm ^−1^)
Cinnamyl alcohol	236.8 ± 9.2	0.47 ± 0.09	0.144 ± 0.009	0.31 ± 0.01
Geraniol	130.9 ± 4.2	0.27 ± 0.04	0.082 ± 0.003	0.29 ± 0.018
Ethanol	142.1 ± 8.8	94.43 ± 8.4	0.089 ± 0.006	(0.94 ± 0.71) × 10^−3^

### Effect of salt and organic solvents on ADH/A1a stability

**Figure 2:**
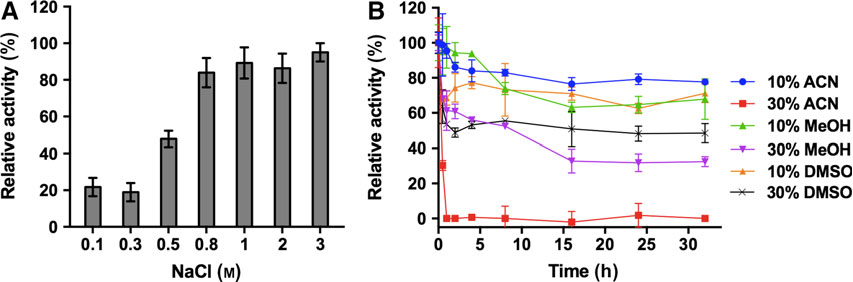
Effect of salt concentration and solvent on the stability of ADH/A1a. (A) Activity of ADH/A1a after 24 h in various salt concentrations. ADH/A1a was dialyzed against varying salt concentrations (10 mm HEPES/NaOH, pH: 7.5, 0.1–3 m NaCl) at 4 °C for 24 h, and the remaining activity was determined. (B) Activity of ADH/A1a over time in different organic solvents. ADH/A1a (in 10 mm HEPES/NaOH, pH 7.5, 2 m NaCl) was incubated with different amounts of organic solvents at room temperature, and the residual activity was assayed over time. ADH/A1a incubated without solvent was treated as the control. The activities of ADH/A1a were assayed in the oxidation reaction under standard conditions. (100% = 0.49 μmol·min^−1^·mg^−1^). Error bars indicate SDs.

Alcohol dehydrogenase/A1a was remarkably stable at high salt concentrations (0.8–3 m NaCl); however, its stability significantly decreased at low salt concentrations (< 0.8 m NaCl) and resulted in loss of activity (Fig. 2A). Though, almost one‐third of the lost activity was regained when dialyzed against the high‐salt buffer (2 m NaCl, 50 mm HEPES/NaOH, pH 7.5).

Importantly, ADH/A1a retained ~ 70–80% of its initial activity after 32 h in 10% (v/v) acetonitrile, methanol, and dimethylsulfoxide (Fig. 2B). At high solvent concentrations (30%, v/v), ADH/A1a was able to withstand dimethylsulfoxide and methanol and retained almost half of its initial activity after 32 h.

### Structural analysis of ADH/A1a

**Figure 3:**
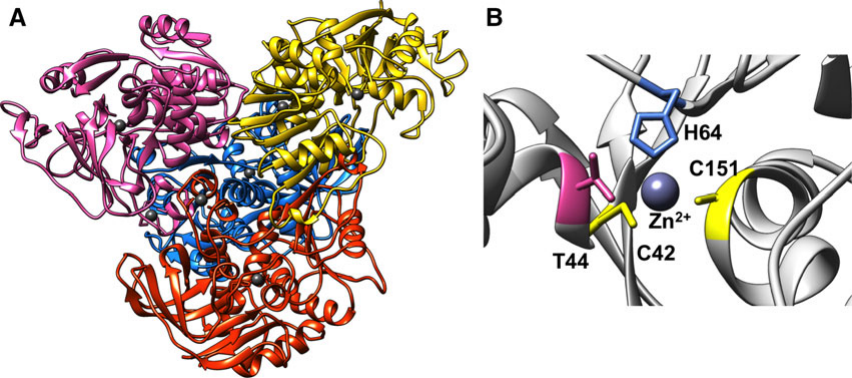
Structure of the homology model of ADH/A1a. (A) Tetrameric structure of ADH/A1a. Monomers are indicated by different colors; Zn ions are dark gray. (B) Conserved catalytic Zn‐binding motif cys42‐his64‐cys151 of ADH/A1a.

The homology model of ADH/A1a was built based on the crystal structure of the thermophilic htADH of *Bacillus stearothermophilus* (PDB: 1rjw, 37% identity) 40. The homology model of ADH/A1a appeared to be reliable, with a QMEAN *Z*‐score of −1.6 and a GMQE of 0.71. High structural conservation was found in the protein core domains, whereas a few less conserved areas were detected in the surface loops, indicating more structural variance (Fig. S10). The quaternary structure of ADH/A1a revealed a homotetramer bearing two conserved zinc‐binding sites per unit (Fig. 3A). The catalytic binding site contains the coordinating residues cys42, his64, and cys151 and resembles the typical binding motif of the zinc‐binding MDR superfamily (Fig. 3B). The structural zinc‐binding site consists of four cysteine residues (cys95, cys98, cys101, and cys109) and is located in the interface region between two monomers. The identified N‐terminal GroES‐like domain encloses the catalytically active center and consists of four β‐strands and a short α‐helix that form a β‐barrel‐like structure 41. Furthermore, the C‐terminal nucleotide‐binding domain is composed of a classical Rossmann fold of six parallel beta‐strands flanked by four alpha‐helices 42.

The thermophilic htADH 40, the mesophilic FurX 43, and the psychrophilic MADH 44 are among the closest homologous structures (Table S3). A comparison with the homology model of ADH/A1a revealed high structural similarities (RMSDs: 0.63–0.7 Å; Fig. S11), although the sequence identity was comparably low (34–37%). Among these homologs, ADH/A1a exhibits the most negative electrostatic surface potential (Fig. S12), resulting from a great abundance of solvent‐exposed acidic residues, particularly of glutamic acid (Figs S13 and S14). Similarly, but to a lesser extent, the cold‐adapted MADH showed an increased negative surface charge, whereby the ratio of glutamic to aspartic acid is balanced. Moreover, both ADH/A1a and htADH exhibited increased amounts of charged surface‐exposed residues, whereas the ratio of hydrophobic residues is reduced (Fig. S13).

### Phylogenetic analysis of ADH/A1a

**Figure 4:**
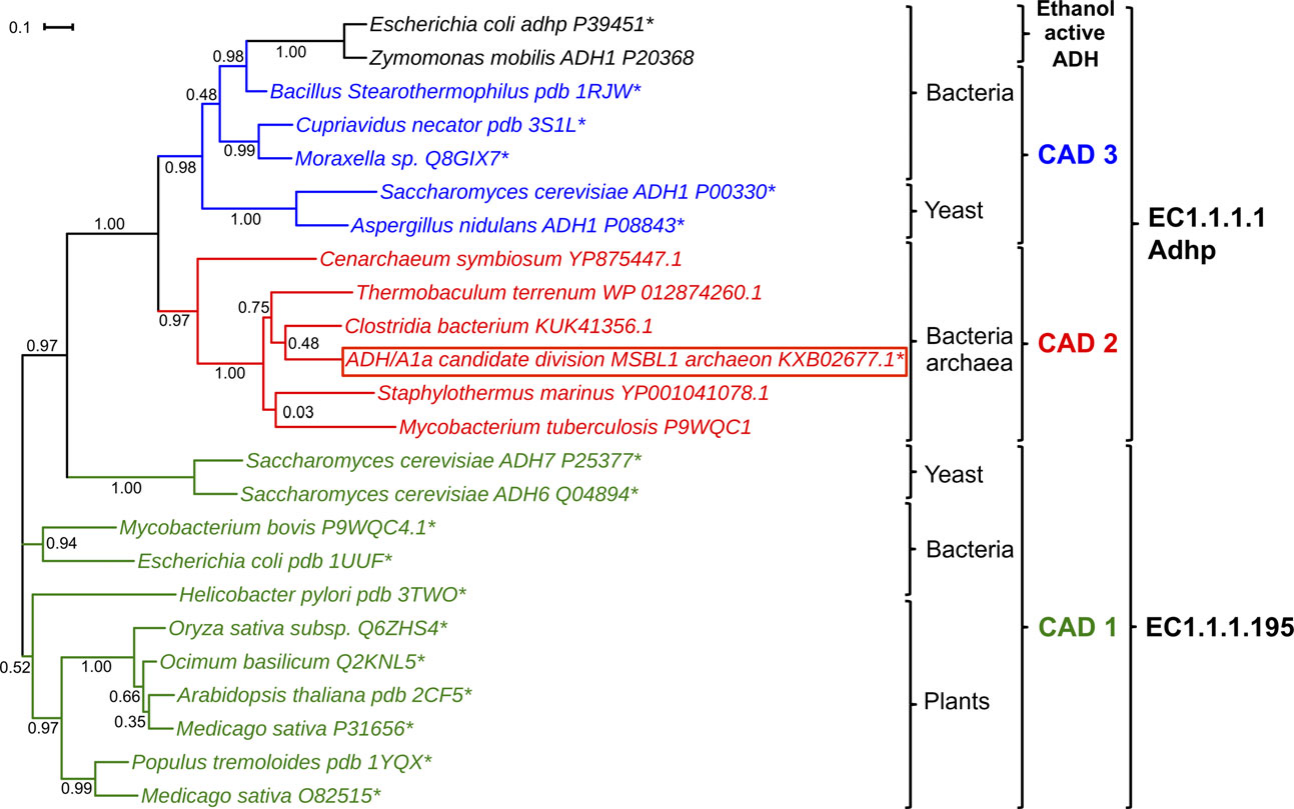
Phylogenetic tree of ADH/A1a and selected ADHs of closest subfamilies. The branches are colored according to the subfamilies, which were annotated using CDD. The EC numbers indicate the functional annotation given by kegg. The calculation of the phylogenetic tree was performed using the maximum‐likelihood algorithm of FastTree2. *Experimentally characterized.

Alcohol dehydrogenase/A1a belongs to the CAD family of the MDRs 15. The CDD divided the CAD family into four subfamilies (CAD1, CAD2, CAD3, and CAD‐like) according to their evolutionarily conserved protein domain patterns by aiming to provide a functional characterization of distinct subfamilies 14. ADH/A1a was annotated as a member of the subfamily CAD2, which has not been investigated to date. However, the functionality‐based annotation by kegg proposed the classification of ADH/A1a as a propanol‐preferring ADH (EC1.1.1.1, AdhP), including several suggested metabolic pathways. Notably, pathways of the classical CADs found in plants (EC1.1.1.195) were not proposed. A sequence alignment of ADH/A1a with classical CADs and with propanol‐preferring ADHs revealed that ADH/A1a shares features with both families (Fig. S15). Interestingly, a few features found in the sequence were unique to the CAD2 subfamily, as an extra loop at position 9–13 and a loop deletion at position 224–225. The structure homologs, htADH, FurX, and MADH, were also classified as propanol‐preferring ADHs (EC1.1.1.1) by kegg, but these ADHs belong to the CAD3 subfamily. CAD3 shares most of its features with ethanol active ADHs and is more closely related to CAD2 than to CAD1, based on our phylogenetic analysis. The phylogenetic tree reveals the positioning of the CAD2 subfamily between the CAD1 and the CAD3/ethanol active ADHs (Fig. 4). We found that the CAD2 subfamily comprises several uncharacterized homologous genes from genome annotations of bacteria and archaea, but not from plants or fungi. The investigation of these CAD2 sequences revealed that most of them were derived from extremophilic environments (Table S4). This evidence corroborates the uniqueness of the CAD2, whose representatives appear like propanol‐preferring ADHs according to kegg, but are able to convert substrates specific for both families, many of them being stable under extremophilic conditions.

## Discussion

The Red Sea brine pools are an attractive source for the discovery of extremozymes. However, microorganisms inhabiting and adapted to these extreme environments are hardly cultivable. With the rise of culture‐independent methods, this ‘microbial dark matter’ is now open for exploration 45. We identified the ADH gene *adh/a1a* by utilizing annotated SAGs, derived from water samples of the Atlantis II Deep Red Sea brine pool 13. Our investigation revealed that ADH/A1a is a polyextremophilic enzyme with special structural stabilities and functionalities, and further gained an understanding of its structural salt and temperature adaptations.

For characterization, we first attempted the expression of ADH/A1a in the mesophilic bacterium *E. coli*. However, ADH/A1a was not successfully expressed since the expression of halophilic enzymes is challenging and generally requires high intracellular salt concentrations 46, 47. Therefore, we used the halophilic *H. volcanii* expression system to express soluble and active ADH/A1a under high‐salt conditions 17.

The extensive characterization of ADH/A1a revealed the halo‐thermophilic character of the enzyme reflected by the activity optima at 4 m salt and 70 °C, resembling the natural conditions of the Atlantis II Deep brine pool. The preference for KCl over NaCl was expected, as KCl is the predominant cytoplasmic ion in cells 48. The pH optima of the reactions performed with ADH/A1a were consistent with several reported ADHs 49. The highly basic pH optimum of the oxidation reaction (pH 10) does not resemble the natural physiological conditions of a cell, but might be explained by the specific thermodynamics of the reaction. The enzyme equivalently converted the cofactors NAD(H) and NADP(H), despite the usual specificity of ADHs for one cofactor 50. ADH/A1a was determined to be zinc dependent and required high zinc concentrations to reach optimal activity. This might be explained by the fact that the natural zinc concentration in the Atlantis II Deep sea brine pool is very high (165.7 μm) compared with normal sea water (0.2 μm) 10. Additionally, zinc ions could bind on the negatively charged surface of ADH/A1a or be involved in folding and structural stabilization of the enzyme 51, 52. Moreover, the his_6_‐tag on ADH/A1a and residual contaminants might bind zinc ions nonspecifically.

Alcohol dehydrogenase/A1a showed remarkable stability in the presence of high salt concentrations and elevated temperatures. However, low‐salt conditions destabilized ADH/A1a, possibly because of the increased structural flexibility and partial unfolding promoted by the decline in external ionic forces 12. Notably, the presence of salt is expected to play an important role in the structural protection of halophilic proteins 53. Furthermore, ADH/A1a was able to withstand exposure to organic solvents. Mesophilic enzymes generally tend to lose their activity in the presence of organic solvents; however, halophilic enzymes are reported to be more stable 8, 54. The stability of ADH/A1a both in solvents and at high temperatures is valuable for biocatalytic reactions in various aspects, for example, increased solubility of organic compounds or improved reaction rates 55. Slower reaction rates were observed compared to the reported homolog ADHs. However, this might be explained by the fact that the life in the extreme of the deep sea brine pools is probably slow and high enzyme activity of ADH is not crucial for survival. The adaptation to the extreme conditions of the habitat might have shifted toward increased stabilities but simultaneously also to reduced activities 21. Furthermore, we cannot exclude that the non‐native reaction environment and the halophilic host expression system might affect the overall native structure and function of the polyextremophilic ADH/A1a, resulting in reduced activity.

Despite the low sequence identity to homologs, we were able to build a reliable ADH/A1a homology model based on the significant structural similarity to the structures of homologous ADHs (RMSD below 1 Å). The enzyme core domains, including the active center and the nucleotide‐binding site, are highly conserved, whereas the solvent‐exposed residues and some flexible regions are altered, enabling the adaptation of the enzyme to the extreme conditions. SWISS‐MODEL calculation and the native PAGE analysis suggest the homotetramer formation of ADH/A1a. Compared to nonhalophilic ADH homologs, the solvent‐exposed surface of the ADH/A1a tetramer displayed increased amounts of glutamic acid residues, resulting in a high negative electrostatic surface potential. A negative surface charge is a known adaptation feature of halophilic enzymes and enables the binding of hydrated ions, which stabilize and increase the solubility of the protein under high‐salt/low‐water conditions 8. A similar adaptation mechanism that enhances enzyme solvation under cold conditions was reported in psychrophilic enzymes, as seen for MADH 56. Furthermore, the surface of ADH/A1a exhibits an increased ratio of charged residues and a decreased ratio of hydrophobic residues, which probably diminishes hydrophobic patches on the surface and resembles another haloadaptive feature 48. An increased ratio of charged amino acids on the surface was also reported for thermophilic enzymes, as we have seen for the homologous htADH 57. The reduction of hydrophobic and the increase of charged residues on the surface add probably to both halo‐ and thermoadaptation.

Alcohol dehydrogenase/A1a effectively oxidized a broad spectrum of alcohols. In the reduction reaction, exclusively cinnamaldehyde, cinnamyl‐methyl‐ketone, and raspberry ketone were reduced. The substrate scope of ADH/A1a coincided with the substrates used by ADHs of the CAD family 58. Based on its conserved domains, ADH/A1a was annotated as a member of the CAD2 subfamily, but its functionality was indicated to be of a propanol‐preferring ADH, rather than of a classical CAD, perhaps due to the lack of experimentally verified CAD2 sequences. Phylogenetic analysis revealed the positioning of ADH/A1a between these ADH families, showing shared features with both, but also unique features specific for the CAD2 family. In plants, the function of CADs in lignin biosynthesis and plant defense mechanisms has been well characterized 59, 60; however, the role of CADs in microorganisms is not yet completely understood. The involvement in several metabolic pathways is considered including the Ehrlich pathway, NAD(P)^+^/H homeostasis, lipid metabolism, amino acid metabolism, and lignin degradation 23, 61, 62. ADH/A1a is the first ADH of the CAD2 subfamily that has been described; thus, its characterization may lead to further understanding of the metabolic functions. Aromatic substrate‐preferring ADHs, such as CADs, are not well investigated to date, despite their great potential in the production of chiral aromatic precursors for pharmaceutical compounds 59, for example, health‐related lignans as podophyllotoxin 63, 64. The substrate‐specific reduction of aromatic ketones combined with the high stability makes ADH/A1a a possible candidate for the production of valuable chiral compounds.

## Authors’ contributions

JE and MR conceived and supervised the study; AA, RK, and JE designed the experiments; AA and RK performed experiments; AA, RK, AH, MV, and IA analyzed the data; SG provided plasmid and support; AA and RK wrote the manuscript; and AH, MV, IA, and SG made manuscript revisions.

## Conflict of interest

The authors declare no conflict of interest.

## Supporting information


**Fig. S1**. Alignment of ADH/A1a and CAD2 consensus sequence.
**Fig. S2.** Size exclusion chromatography (SEC) purification of ADH/A1a.
**Fig. S3.** Coomassie‐stained SDS/PAGE of purified ADH/A1a.
**Fig. S4**. Coomassie‐stained native PAGE of purified ADH/A1a.
**Fig. S5.** Tryptic digest and LC‐MS/MS analysis of ADH/A1a.
**Fig. S6.** Measured melting curve and fitting curve.
**Fig. S7.** Calculated free Gibbs energy Δ_r_ *G*′ at different pH.
**Fig. S8**. Effect of various parameters on the ADH/A1a activity.
**Fig. S9**. Michaelis–Menten kinetic of various substrates.
**Fig. S10**. Quality assessment of the homology model.
**Fig. S11.** Alignment of tetrameric structures of ADH/A1a and homologs.
**Fig. S12.** Electrostatic surface charge of ADH/A1a tetramer and structure homologs.
**Fig. S13.** Composition of surface‐exposed amino acids of ADH/A1a and homologs.
**Fig. S14.** Ratio of amino acids on the solvent‐exposed surface.
**Fig. S15**. Sequence alignment of selected ADHs of the CAD1, CAD2, CAD3 subfamily and propanol‐preferring ADHs.
**Table S1.** Identified contaminant proteins of the host strain.
**Table S2.** ICP‐OES measurement of zinc concentration of an ADH/A1a solution.
**Table S3.** Information of closest homologous ADHs with known crystal structures.
**Table S4.** Information of closest ADHs based on amino acid sequence.
