# Supplementary material for: A polyextremophilic alcohol dehydrogenase from the Atlantis II Deep Red Sea brine pool
Source: FEBS Open Bio. 2018 Dec 18;9(2):194–205. doi: 10.1002/2211-5463.12557 (PMC6356862; doi:10.1002/2211-5463.12557)
Supplement: Supplementary file 1 — Fig. S1. Alignment of ADH/A1a and CAD2 consensus sequence. Fig. S2. Size exclusion chromatography (SEC) purification of ADH/A1a. Fig. S3. Coomassie‐stained SDS/PAGE of purified ADH/A1a. Fig. S4. Coomassie‐stained native PAGE of purified ADH/A1a. Fig. S5. Tryptic digest and LC‐MS/MS analysis of ADH/A1a. Fig. S6. Measured melting curve and fitting curve. Fig. S7. Calculated free Gibbs energy Δr G′ at different pH. Fig. S8. Effect of various parameters on the ADH/A1a activity. Fig. S9. Michaelis–Menten kinetic of various substrates. Fig. S10. Quality assessment of the homology model. Fig. S11. Alignment of tetrameric structures of ADH/A1a and homologs. Fig. S12. Electrostatic surface charge of ADH/A1a tetramer and structure homologs. Fig. S13. Composition of surface‐exposed amino acids of ADH/A1a and homologs. Fig. S14. Ratio of amino acids on the solvent‐exposed surface. Fig. S15. Sequence alignment of selected ADHs of the CAD1, CAD2, CAD3 subfamily and propanol‐preferring ADHs. Table S1. Identified contaminant proteins of the host strain. Table S2. ICP‐OES measurement of zinc concentration of an ADH/A1a solution. Table S3. Information of closest homologous ADHs with known crystal structures. Table S4. Information of closest ADHs based on amino acid sequence. [file FEB4-9-194-s001.pdf]

Supporting information

A polyextremophilic alcohol dehydrogenase from the Atlantis II Deep Red Sea brine pool

Anastassja L. Akal[1,2], Ram Karan[1], Adrian Hohl[1,2], Intikhab Alam[3], Malvina Vogler[1,2], Stefan Groetzinger[3,4], Jörg Eppinger[1], Magnus Rueping[1,5*]

[1] King Abdullah University of Science and Technology (KAUST), KAUST Catalysis Center (KCC), Thuwal 23955-6900, Saudi Arabia

[2] Technical University of Munich (TUM), Center for Integrated Protein Science Munich at the Department of Chemistry, Garching 85748, Germany

[3] King Abdullah University of Science and Technology (KAUST), Computational Bioscience Research Center (CBRC), Thuwal 23955-6900, Saudi Arabia

[4] Technical University of Munich (TUM), Institute of Biochemical Engineering, Garching 85748, Germany

[5] RWTH Aachen, Institute of Organic Chemistry, Aachen 52074, Germany

[*] Corresponding author

Magnus Rueping: magnus.rueping@kaust.edu.sa

Phone number: +966 12 8087346

INDEX

I. Sequence analysis and alignment of ADH/A1a and CAD2 consensus sequence

II. Analysis of purified ADH/A1a by SDS-PAGE

III. Analysis of ADH/A1a by native PAGE

IV. Tryptic digest and LC-MS/MS analysis of ADH/A1a

V. Thermal stability of ADH/A1a

VI. Thermodynamic analysis of the reactions using eQuilibrator

VII. Zinc content of active ADH/A1a solution

VIII. Effect of various parameters on the ADH/A1a activity

XI. Michaelis-Menten kinetic of various substrates

X. Quality assessment of the homology model

XI. Comparison of homologous crystal structures with the ADH/A1a model

XII. Distribution of surface exposed amino acids of ADH/A1a and homologues.

XIII. Sequence alignment of selected ADHs from different CAD subfamilies

XIV. Information of closest homologous ADHs

I. Sequence analysis and alignment of ADH/A1a and CAD2 consensus sequence

```
                                                      vvv
ADH/A1a      MKAMQLKEPKPVEQAPLEMVELKEPRPGPKEVKIDVQACGVCHTDLHTVEGELSLPKLPI  60
CAD2         MKAMVLEKPGPIEENPLRLTEVPVPEPGPGEVLIKVEACGVCRTDLHIVEGDLPPPKLPL
             **** *::* *:*: **.:.*:  *.*** ** *.*:*****:**** ***:*. ****:

                v                         v    v   v  v       v
ADH/A1a      VPGHEVVGVVEESGDEAEHFEVGDRVGVTWLYSSCGECKFCRRGQENLCEDPMFTGLHAD 120
CAD2         IPGHEIVGRVEAVGPGVTRFSVGDRVGVPWLGSTCGECRYCRSGRENLCDNARFTGYTVD
             :****:** **  *  . :*.*******.** *:****::** *:****::. ***  .*

                                           v                       vvvvvv
ADH/A1a      GGYEESMVAKEDFVYPIPKNISDEDAAPLLCAGVIGYRSLRLSEVKPGQRLGLFGFGASA 180
CAD2         GGYAEYMVADERFAYPIPEDYDDEEAAPLLCAGIIGYRALKLAGLKPGQRLGLYGFGASA
             *** * ***.* *.****:: .**:********:****:*:*: :********:******

                              vv       v      v                  vv  v
ADH/A1a      HIVIQLATDMGCEVYVFTRSEEHRRLARELGSAWEGSAKDDPPHRIDSGITFAPVGWIVK 240
CAD2         HLALQIARYQGAEVFAFTRSGEHQELARELGADWAGDSDDLPPEPLDAAIIFAPVGALVP
             *:.:*:*   *.**:.**** **:.******: * *.:.* **. :*:.* ***** :*

                           vv                        vvv
ADH/A1a      EALRDLEKGGTLAINAIHMTPIPELDYDLIYHEKKLRSVANVTREDAEGFLKIAGDIPVQ 300
CAD2         AALRAVKKGGRVVLAGIHMSDIPAFDYELLWGEKTIRSVANLTRQDGEEFLKLAAEIPIK
              *** ::*** :.: .***: ** :**:*:: **.:*****:**:*.* ***:*.:**::

ADH/A1a      TEVETFPLEEANRALRLLKDSKINGAGVLKVS 332
CAD2         PEVETYPLEEANEALQDLKEGRIRGAAVL---
             .****:******.**: **:.:*.**.**
```

v - Catalytical Zn binding site

v - Structural Zn binding site

v - putative NAD(P) binding site

v - putative substrate binding site

beta sheet

alpha helix

0 1 – 152 GroES-like domain

153 – 294 NAD(P)-binding domain

Fig. S1. Alignment of ADH/A1a and CAD2 consensus sequence. Conserved subdomains are highlighted. Predicted binding sites are marked. Secondary structure elements of ADH/A1a were predicted using JPred and are shown below the corresponding amino acid sequence. CAD2 consensus sequence was derived from the CDD.

II. Analysis of purified ADH/A1a by SDS-PAGE

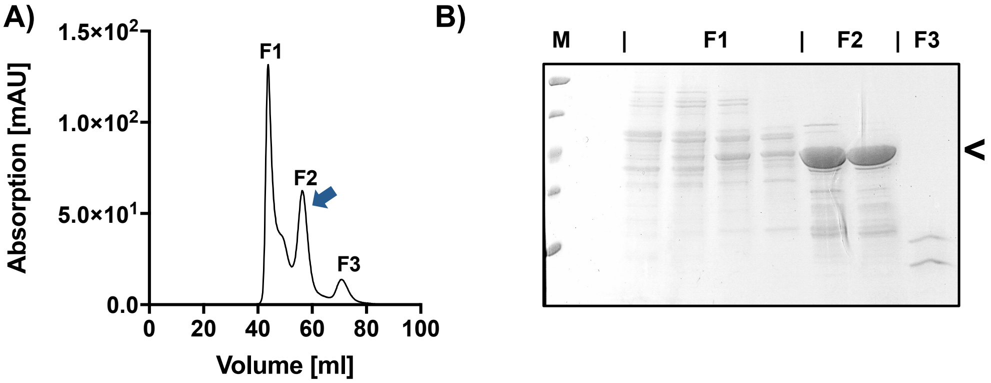

Fig. S2. Size exclusion chromatography (SEC) purification of ADH/A1a. (A) Chromatogram of the size exclusion chromatography (SEC); (B) Coomassie-stained SDS-PAGE of collected fractions of SEC purification. ADH/A1a is visible at ~40 kDa in fraction F2. The fractions of peak F2 were pooled and concentrated.

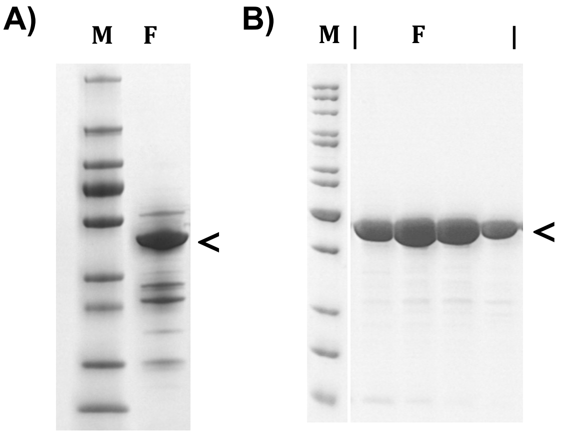

Fig. S3. Coomassie-stained SDS-PAGE of purified ADH/A1a. The main band of ADH/A1a is visible at ~40 kDa; (A) The protein was purified by IMAC followed by a SEC at high salt concentrations (2 M NaCl) including 10 % (v/v) glycerol. The purified protein was active. (B) The protein was purified using IMAC at low salt concentrations (0.5 M NaCl). The purified protein was inactive. (M) protein ladder; (F) fractions.

III. Analysis of ADH/A1a by native PAGE

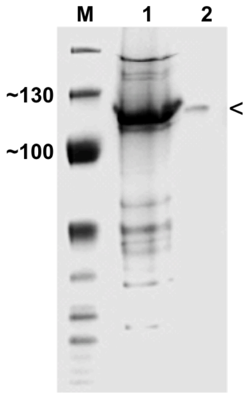

Fig. S4. Coomassie-stained native PAGE of purified ADH/A1a. (M) Mesophilic protein ladder, (1) ADH/A1a (high conc.), (2) ADH/A1a tetramer (low conc.); conditions: sample loading buffer: 50 mM Tris-HCl pH 6.8, 1 M NaCl, 10% (w/v) sucrose, 0.003% bromophenol blue; running buffer: 25 mM Tris, 192 mM glycine, pH 8.3.

The native PAGE analysis of ADH/A1a showed a prominent band corresponding to about 120 kDa, indicating that this enzyme is oligomeric (Fig S4). However, in case of the halophilic ADH/A1a, we could not accurately determine the native molecular weight by native PAGE, as the mobility of the halophilic enzyme might be influenced by the increased acidic charge (pI ~ 5) and the high salt concentration of the sample.

IV. Tryptic digest and LC-MS/MS analysis of ADH/A1a

1 MHHHHHHGSG LKAMQLK**EPK PVEQAPLEMV ELK**EPRPGPK EVK**IDVQACG**
51 VCHTDLHTVE GELSLPKLPI VPGHEVVGVV EESGDEAEHF EVGDRVGVTW
101 LYSSCGECKF CR**RGQENLCE DPMFTGLHAD GGYEESMVAK EDFVYPIPKN**
151 ISDEDAAPLL CAGVIGYRSL RLSEVKPGQR LGLFGFGASA HIVIQLATDM
201 GCEVYVFTRS EEHRRLAR**EL GSAWEGSAK**D DPPHR**IDSGI TFAPVGWIVK**
251 EALRDLEK**GG TLAINAIHMT PIPELDYDLI YHEK**KLRSVA NVTR**EDAEGF**
301 LKIAGDIPVQ TEVETFPLEE ANRALRLLKD SK**INGAGVLK** VS

Fig. S5. Tryptic digest and LC-MS/MS analysis of ADH/A1a. The protein was exercised from a SDS-PAGE band corresponding to the size of ADH/A1a. Sequence coverage: 66 %, matched peptides shown in ***bold.***

Table S1: Identified contaminant proteins of the host strain.

Sample	Contaminant protein	Seq. coverage
**1**	Proteasome-activating nucleotidase 1<br>Organism: *Hfx. volcanii*	41%
**2**	50S ribosomal protein<br>Organism: *Hfx. volcanii*	24%
**3**	Sec-independent protein translocase protein TatAo<br>Organism: *Hfx. volcanii*	23%

Contaminant protein bands were identified by tryptic digest.

V. Thermal stability of ADH/A1a

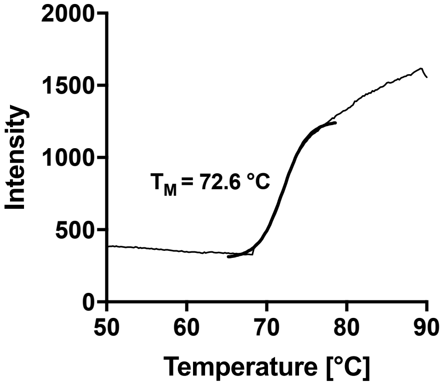

Fig. S6. Measured melting curve and fitting curve; fair line: measured melting curve, bold line: fitted curve.

VI. Thermodynamic analysis of the reactions using eQuilibrator

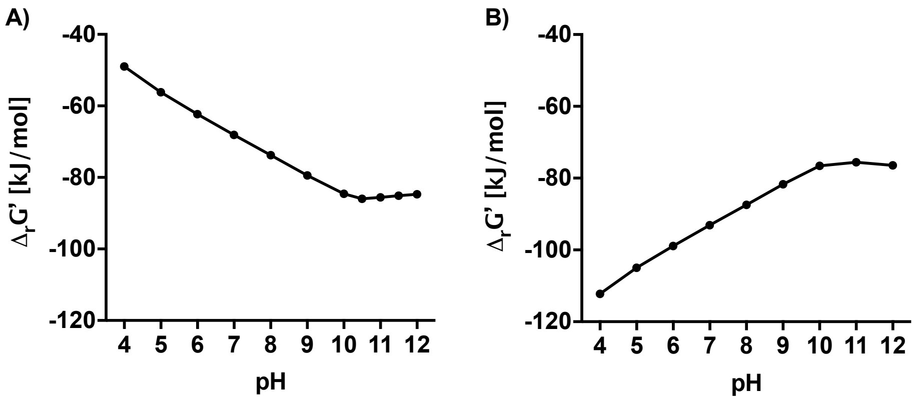

Fig. S7. Calculated free Gibbs energy $\Delta_r G'$ at different pH. (A) Oxidation reaction of cinnamyl alcohol (15.5 mM) with NAD^+ (10 mM); (B) Reduction reaction of cinnamyl aldehyde (31 mM) with NADH (0.2 mM). An ionic strength of 3 M was applied. The values were calculated using eQuilibrator.

VII. Effect of various parameters on the ADH/A1a activity

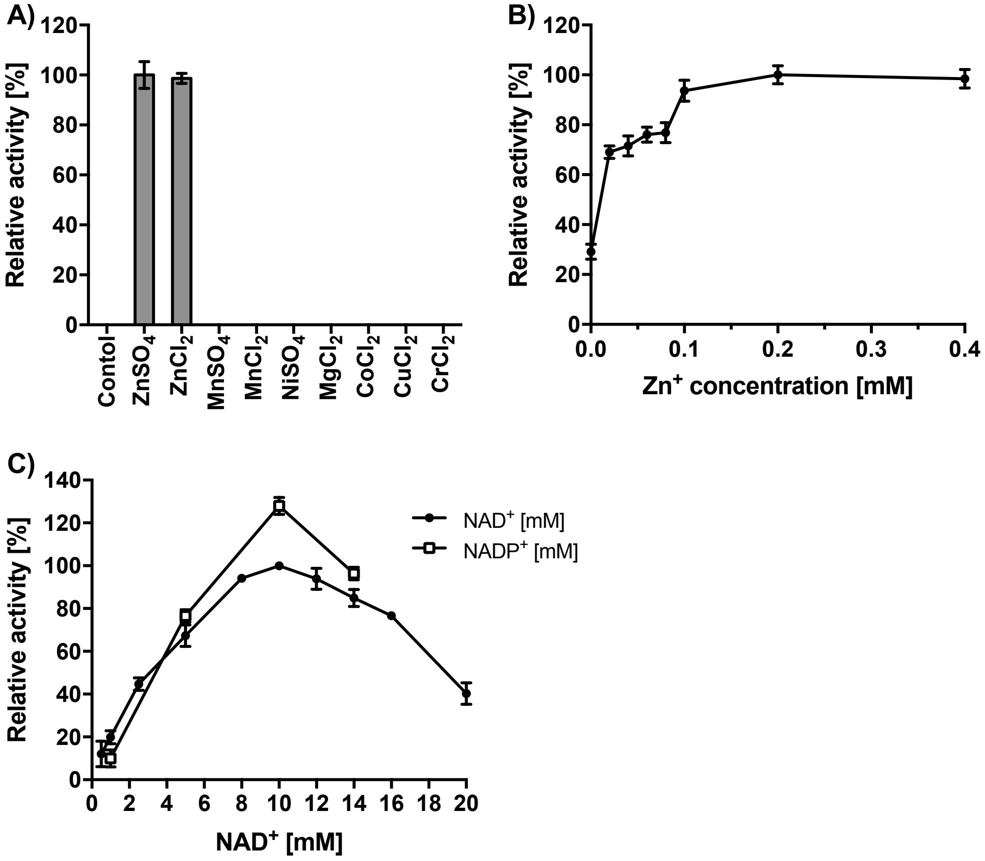

Fig. S8. Effect of various parameters on the ADH/A1a activity. (A) Metal-free ADH/A1a incubated with different metal ions. As control metal-free ADH/A1a without additive was used; (B) different zinc sulfate concentrations; (C) different NAD^+ and $NADP^+$ concentrations. Error bars indicate SDs.

VIII. Zinc content of active ADH/A1a solution

Table S2 - ICP-OES measurement of zinc concentration of an ADH/A1a solution.

Sample	Condition	Measured zinc concentration
**ADH/A1a**	ADH/A1a (44.64 μM)	55.4 ppm ± 2.75 ppm
**(+) control**	buffer with 0.1 mM zinc sulfate	6.09 ppm ± 0.52 ppm
**(−) control**	Buffer (20mM HEPES, pH 7.5, 2M NaCl)	0.04 ppm ± 0.00 ppm

IX. Michaelis-Menten kinetic of various substrates

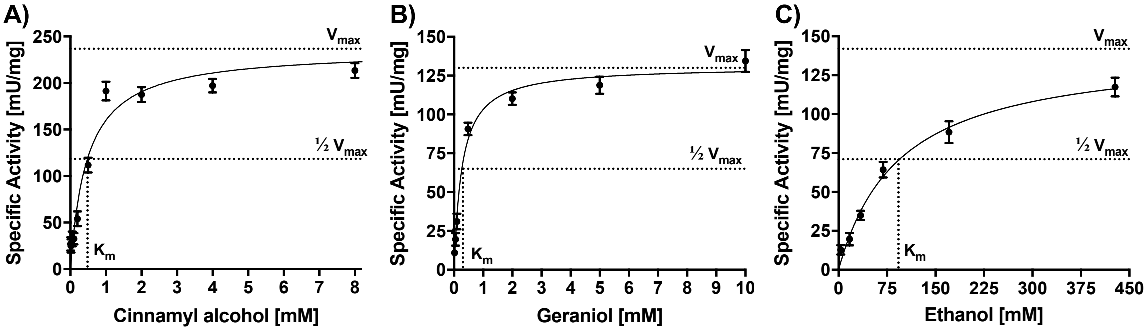

Fig. S9. Michaelis-Menten kinetic of various substrates. (A) cinnamyl alcohol, (B) geraniol and (C) ethanol. Enzymatic activities were measured using standard conditions. Error bars indicate SDs.

X. Quality assessment of the homology model

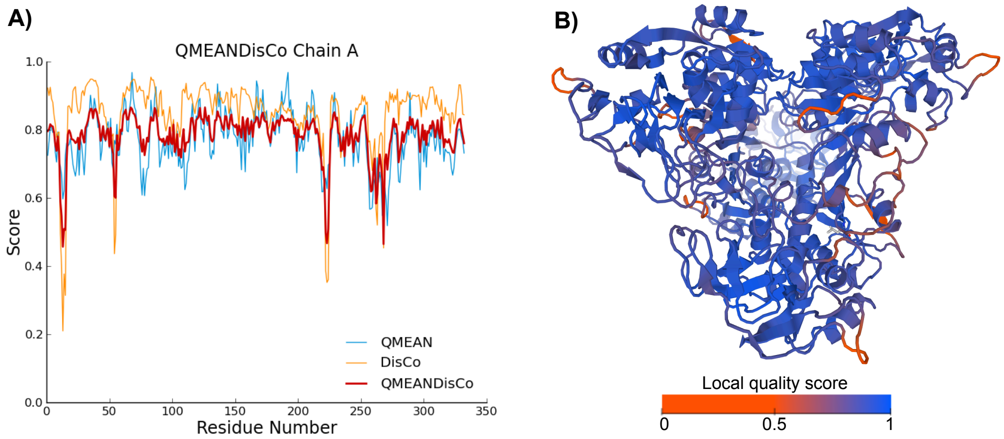

Fig. S10. Quality assessment of the homology model. (A) Local quality scores are shown for each amino acid position of the monomer chain A. The overall quality of the homology model is reliable (scores around 0.8), except of some more flexible positions (scores < 0.6). QMEAN: Quality model energy analysis; Disco: distance constraint score; QMEANDisCo: local quality score, merged scores of QMEAN and Disco (values between 0 and 1, values below 0.6 are of low quality). (B) Local quality scores shown on the structure model. Pictures were derived using the QMEANDisco tool from the QMEAN server [1].

XI. Comparison of homologous crystal structures with the ADH/A1a model

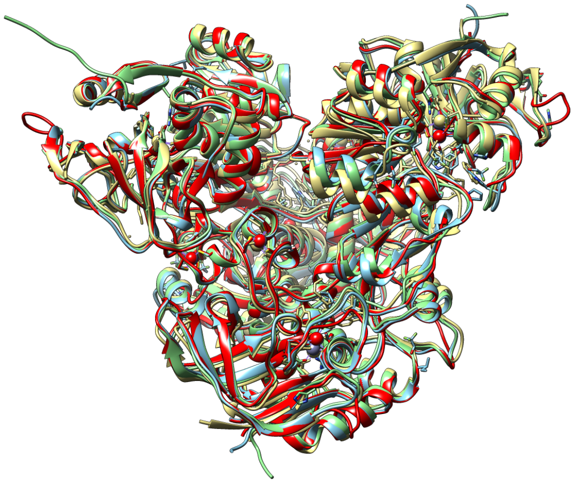

Fig. S11. Alignment of tetrameric structures of ADH/A1a and homologs. The alignment of the structures was done using UCSF Chimera [2]; red: ADH/A1a, light blue: htADH, light yellow: FurX, light green: MADH.

XII. Distribution of surface exposed amino acids of ADH/A1a and homologs.

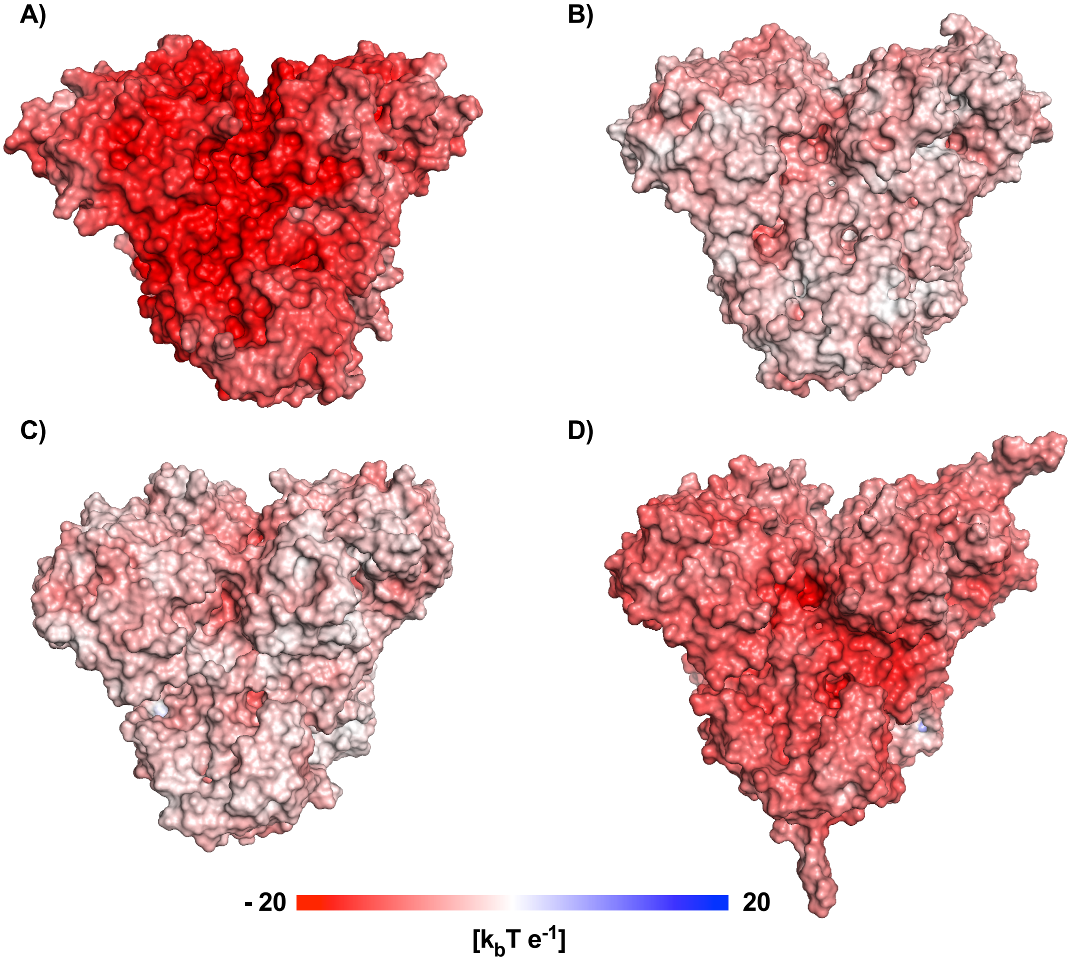

Fig. S12. Electrostatic surface charge of ADH/A1a tetramer and structure homologs. Charged amino acid residues are colored from red (negative) to blue (positive). (A) Homology model of ADH/A1a; (B) thermophilic htADH (PDB: 1rjw) from *Bacillus stearo-thermophilus* (RMSD 0.63 Å) [3]; (C) mesophilic FurX (PDB: 3s1l) from *Cupriavidus necator* (RMSD 0.7 Å) [4]; (D) psychrophilic MADH (4z6k) from *Moraxella sp.* (RMSD 0.68 Å) [5]. Unit: k_b= Boltzmann constant, T = temperature [K], e = charge of an electron.

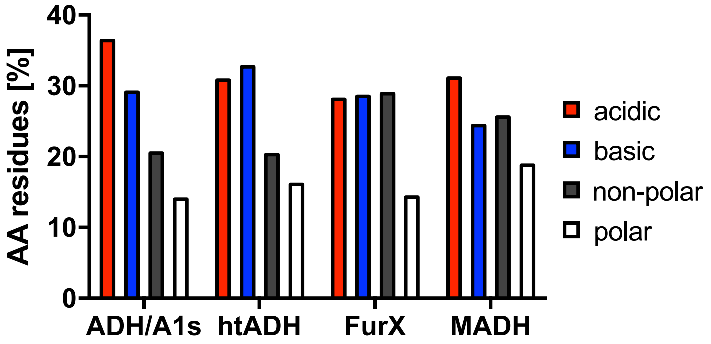

Fig. S13. Composition of surface-exposed amino acids of ADH/A1a and homologs. The ratio of acidic, basic, polar, and non-polar amino acids from the tetramer surface. The homologous ADHs are thermophilic htADH (PDB: 1rjw), mesophilic FurX (PDB: 3s1l), and psychrophilic MADH (PDB: 4z6k).

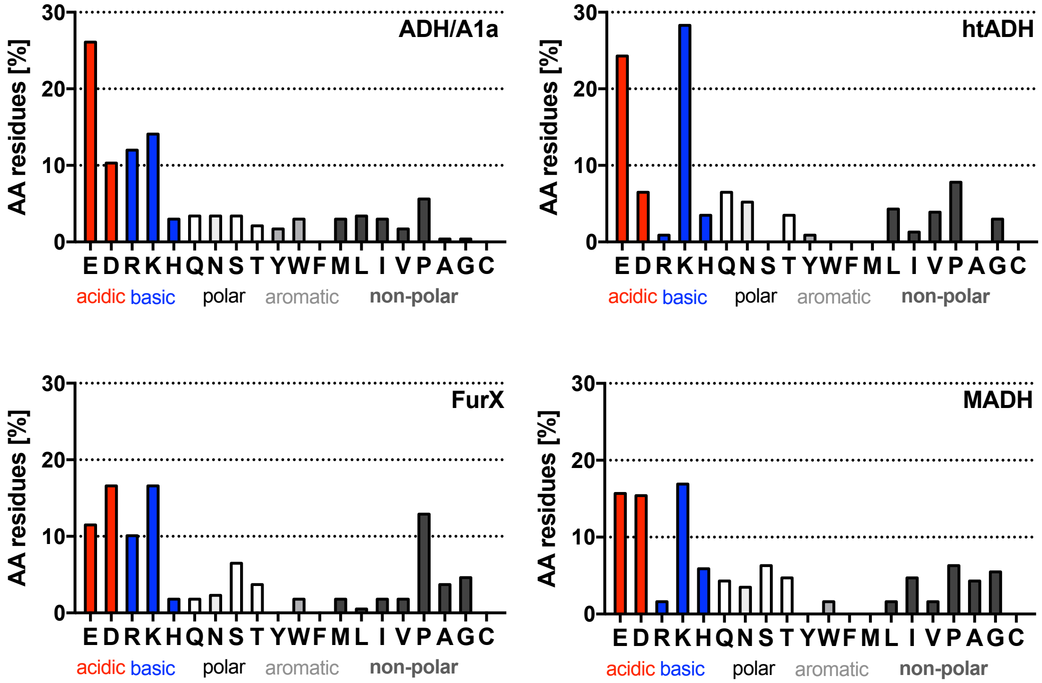

Fig. S14. Ratio of amino acids on the solvent-exposed surface, determined using Swiss PDB viewer 4.1.1 with a surface accessibility of ≥ 30%.

XIII. Sequence alignment of selected ADHs from different CAD subfamilies

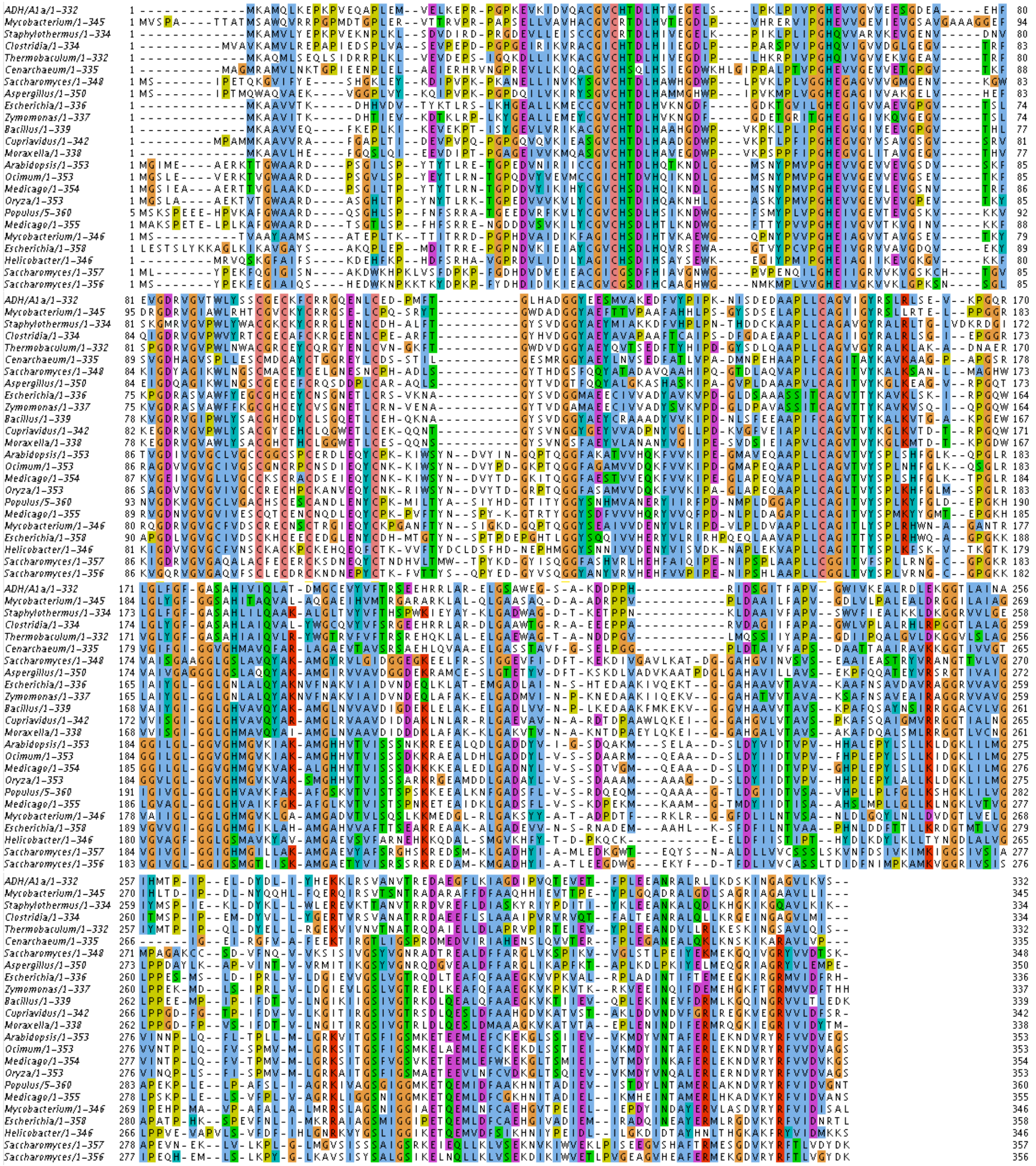

Fig. S15. Sequence alignment of selected ADHs of the CAD1, CAD2, CAD3 subfamily and propanol-preferring ADHs. The alignment was used for the phylogenetic tree (Fig. 7). The alignment was done using MAFFT and illustrated using Jalview 1.0 [6]. The coloring of the amino acids is according to the Clustal coloring scheme. Blue – hydrophobic, red – positive charge, magenta – negative charge, green – polar, pink – cysteines, orange – glycines, yellow- prolines, cyan – aromatic, white – unconserved.

XIV. Information of closest homologous ADHs

Table S3. Information of closest homologous ADHs with known crystal structures

Name and PDB ID	Organism	Type	Swiss-model rank	I-Tasser rank	RMSD [Å]	Seq. ident. [%]	Ref.
htADH 1rjw	*Bacillus stearo-thermophilus*	thermophile	1	1	0.63	37	[3]
FurX 3s11	*Cupriavidus necator*	mesophile	2	3	0.70	36	[4]
MADH 4z6k	*Moraxella sp.* TAE123	psychrophile	4	2	0.68	34	[5]

The ADH/A1a homologs were found using the sequence-based prediction approaches Swiss-model and I-Tasser. The rankings of the structures are shown for both approaches as well as the sequence identity to ADH/A1a. The route mean square deviation (Rmsd) corresponds to the differences between the amino acid residues that are structurally aligned by TM-align of I-Tasser. All homologous ADHs incorporated two zinc(II) ions per monomer and used NAD(H) as cofactor.

Table S4. Information of closest ADHs based on amino acid sequence

Accession no	Organism	Sampling site and conditions	Seq. Ident.
XA89544.1	*Candidate divison MSBL1 archaeon*	Red Sea Discovery brine pool, 44.8 °C, 26.2% salt, pH 6.2, 2141 m	79%
OGL42381.1	*Candidatus Schekmanbacteria bacterium*	Suboxic/anoxic aquifer, groundwater, Colorado USA, 5 m, pH 7.2	61 %
CBE70068.1	*Candidatus Methylomirabilis oxyfera*	Several ecosystems, including soil, sediments, groundwater and marine habitates, methanotroph	61 %
WP_015739180.1	*Ammonifex degensii*	Neutral volcanic hot spring, 70 °C, 0.1 M NaCl	61 %
OUC09238.1	*Litorilinea aerophila*	Intertidal hot spring, Iceland 72 °C, 0.6% salt	60 %
KUK41356.1	*Clostridium sp.**	Oil reservoir, Kuparuk, Alaska North Slope, 65 - 80 °C, 2.1% salt	60 %

WP_014434993.1	*Caldilinea aerophila*	Japanese hot spring, 55 °C, pH 7.5	59 %
WP_014808123.1	*Desulfomonile tiedjei*	Municipal digester sludge from Adrian, Michigan, 20 - 38 °C, pH 6.5 - 7.8	56 %
WP_016482519.1	*Chthonomonas calidirosea*	Geothermal heated soil, New Zealand, 55 °C, pH 4.3	56 %
WP_082726423.1	*Limnochorda pilosa*	Sediment of a brackish meromictic lake, 45 - 50 °C, pH 7.0	55 %
WP_012874260.1	*Thermobaculum terrenum**	Thermal soil, Yellowstone Park, pH 3.9, 65 - 92 °C	54 %
WP_011697699.1	*Syntrophobacter fumaroxidans*	Anaerobic, 37 ˚C	54 %
WP_080885594.1	*Nitrospira japonica*	Wastewater treatment plant	52 %
YP_002753716.1	*Acidobacterium capsulatum*	Acidophilic, chemoautotrophic, mesophilic	52 %

Based on the amino acid sequence of ADH/A1a sequential homologues were searched in the database of NCBI and KEGG. * appearing in the phylogenetic tree.

References

1. Benkert P, Kunzli M, and Schwede T (2009) QMEAN server for protein model quality estimation. *Nucleic Acids Res* **37**, W510-514.
2. Pettersen EF, Goddard TD, Huang CC, Couch GS, Greenblatt DM, Meng EC, and Ferrin TE (2004) UCSF Chimera--a visualization system for exploratory research and analysis. *J Comput Chem* **25**, 1605-1612.
3. Ceccarelli C, Liang Z-X, Strickler M, Prehna G, Goldstein BM, Klinman JP, and Bahnson BJ (2004) Crystal structure and amide H/D exchange of binary complexes of alcohol dehydrogenase from *Bacillus stearothermophilus*: insight into thermostability and cofactor binding. *Biochemistry* **43**, 5266-5277.
4. Kang C, Hayes R, Sanchez EJ, Webb BN, Li Q, Hooper T, Nissen MS, and Xun L (2012) Furfural reduction mechanism of a zinc-dependent alcohol dehydrogenase from *Cupriavidus necator* JMP134. *Mol Microbiol* **83**, 85-95.
5. Tsigos I, Velonia K, Smonou I, and Bouriotis V (1998) Purification and characterization of an alcohol dehydrogenase from the Antarctic psychrophile *Moraxella sp.* TAE123. *Eur J Biochem* **254**, 356-362.
6. Clamp M, Cuff J, Searle SM, and Barton GJ (2004) The Jalview Java alignment editor. *Bioinformatics* **20**, 426-427.
